# Abstracts DGKCH

**DOI:** 10.1515/iss-2019-2004

**Published:** 2019-03-20

## DGKCH/DGNC: Brain malformations: Diagnosis and Therapy

### Fetoscopic coverage of myelomeningoceles in spina bifida creates a new commodity of patients

(Abstract ID: 160)

T. Fortmann^1^, A. Brentrup^2^

^1^*Hamm*

^2^*Uniklinik Münster*

**Background:**

After proving the feasability of intrauterine closure of spina bifida defects in sheep in 2003 the university hospital of Gießen and Marburg started the percutaneous fetoscopic patch coverage of spina bifida aperta. Due to this new kind of surgery a new commodity of patients developed. These patients have different problems than postnatally operated spina bifida patients.

**Materials and methods:**

We report of our experiences in Muenster of eight patients who present themselves with different kind of problems.

**Results:**

Wound healing is a big issue for these patients. Two patients underwent postpartal surgery due to dural tears, one twice. Two patients had local infections resulting in prolonged wound healing. One patient developed a secondary meningomyelocele and needed open surgery.

The anatomy of the celes is not respected as in postpartal surgery. This means that in case of revision surgery, it is difficult to separate adequate layers of tissue for covering the defects. Two patients have a tethered cord.

One patient has a remaining anchor, which makes MRI imaging difficult/impossible.

Chiari malformations are not prevented by fetoscopic coverage - one patient even needed decompression.

Six patients still developed a hydrocephalus and needed a shunt supply.

**Conclusion:**

From our experience in Muenster a new commodity of patients developed after the beginning of fetoscopic coverage of spina bifida aperta, which needs special attention due to a difficult wound healing, a different postsurgical anatomy and so far uncommon problems for spina bifida patients.

### Clinical Outcome after Decompression in Chiari Malformation

(Abstract ID: 436)

A. Peraud^1^, N. Goldschagg^2^, M. Kunz^2^, J.-C. Tonn^2^, M. Strupp^2^

^1^*Universitätsklinikum Ulm, Ulm*

^2^*Uniklinik München*

**Background:**

Treatment of Chiari malformation includes suboccipital decompression with dural enlargement and if necessary resection of one cerebellar tonsil. The effect of tonsillar resection on ocular motor and cerebellar function have not yet been systematically examined. The purpose of this study was to investigate whether decompression, including resection of one cerebellar tonsil, leads to ocular motor, vestibular, or cerebellar deficits.

**Materials and methods:**

Ten patients with Chiari malformation type I were systematically examined before and after (1 week and 3 months) suboccipital decompression with unilateral tonsillectomy. The work-up included a neurological and neuro-ophthalmological examination, vestibular function, posturography, and subjective scales. Cerebellar function was evaluated by ataxia rating scales.

**Results:**

Suboccipital decompression with tonsillar resection resulted in a significant subjective improvement 3 months after surgery with regard to headache (5/5 patients), hyp-/dysesthesia (5/5 patients), ataxia of the upper limbs (4/5 patients), and paresis of the triceps and interosseal muscles (2/2 patients). Ocular motor disturbances before decompression were detected in 50% of the patients. These symptoms improved after surgery, but five patients had new persisting mild ocular motor deficits 3 months after decompression with unilateral tonsillectomy (i.e., smooth pursuit deficits, horizontally gaze-evoked nystagmus, rebound, and downbeat nystagmus) without any subjective complaints. Impaired vestibular (horizontal canal, saccular, and utricular) function improved in five of seven patients with impaired function before surgery. Posturographic measurements after surgery did not change significantly.

**Conclusion:**

Decompression, including resection of one cerebellar tonsil, leads to an effective relief of patients’ preoperative complaints. It is a safe procedure when performed with the help of intraoperative electrophysiological monitoring, although mild ocular motor dysfunctions were seen in half of the patients, which were fortunately asymptomatic.

### Ventriculoperitoneal shunt versus endoscopic third ventriculostomy in children less than one year: A single center report

(Abstract ID: 544)

S. Deininger^1^

^1^*Universitätsklinik Ulm*

**Background:**

Endoscopic third ventriculostomy (ETV) is established as a treatment option in children with hydrocephalus. Age and etiology are considered to be main factors influencing success of ETV in children. In literature ETV seems to be associated with less revisions compared to VP-shunting. However the success rate in children less than 1 year of age is worse than in older children, as they are considered to be high risk patients.

**Materials and methods:**

In a matched-pair analysis we retrospectively compared two groups of children (n=49). Children < 1 year of age to a maximum of 11kg undergoing primary ETV or VP-shunting were included in the study. In all patients etiology was a different type of obstructive hydrocephalus. In VP-shunted patients a programmable (Medos Hakim®) shunt valve was implanted. During ETV procedure a Rickham Reservoir was applied for possible emergency drainage. Primary endpoints were time to failure (1), number of revisions (2) and patency (3) within a period of 18 months of observation. Failure was defined as the need for shunt revision, placement of a new shunt or re-ventriculostomy.

**Results:**

In our patients obstructive etiologies most frequent were aqueductal stenosis following intraventricular hemorrhage (IVH) or due to Chiari malformations. The percentage of preterm babies was 37%.

Mean age of VP-shunted children was 66 days, 14/26 (54%) children did not require a second procedure within time of observation. In those patients requiring secondary surgery mean time to revision was 160 days. Children after primary VP-shunting undergoing secondary surgery obtained 2 revisions in average.

In the group of children treated primarily with ETV mean age was 68 days. 8/23 (35%) children did not require a second surgical intervention. For patients with secondary surgery time to revision was shorter compared to VP-shunting with being 85 days in average, while the average number of revisions was 2.

**Conclusion:**

In our patient population ETV and VP-shunting showed similar results after 18 months, however 35% of children after ETV could remain free of a shunt valve.

### Deafness due to Inner Ear Malformations is successfully treated by Auditory Brainstem Implantation (ABI) in Prelingually deaf children

(Abstract ID: 743)

R. Behr^1^, K. Schwager^1^, E. Hofmann^1^

^1^*Klinikum Fulda*

**Background:**

Children born with severe cochlear malformation, cochlear aplasia or hypoplasia/aplasia of the cochlear nerve are no good candidates for cochlear implantation (CI). The only possibility for restoration of hearing in these cases is direct stimulation of the hearing pathways at brainstem level.

**Materials and methods:**

A 12 channel ABI system (MedEl, Austria), which has demonstrated effectiveness and safety in neurofibromatosis patients since the last 21 years was used for implantation. The pediatric ABI program started in 2009. Meanwhile 38 implantations in 37 children were performed by the first author. Three children had a successful revision surgery after a fall on the implant side, a dislocation of the probe and a spontaneous breakdown of an other ABI system. The mean age was 3.3 years, median 2.8. The youngest was 1.25, the oldest 6.5 yrs. Surgery was performed in supine position using a retrosigmoid approach and multimodal neuromonitoring. In all cases intraoperative E-BERA were recorded.

**Results:**

The preoperative evaluation with high resolution MRI and CT revealed in 26 children aplasia of the cochlear nerve. The others had cochlear dys-aplasia together with hypoplasia of the 8th nerve or syndromal lesions like Goldenhar or CHARGE Syndrome. In most cases surgery was difficult due to complete or partial occlusion of the lateral recess of the forth ventricle ( foramen of Luschkae). In 75% branches of the AICA or the vessel itself were crossing the implant site and had to be dissected. In every case the electrode paddle was small enough to fit properly into the recess. E-BERA recordings could be derived in each case. There were no neurologic complications and only minor surgical complications as subcutaneous CSF leaks in 7 children. All children in whom the device was activated so far regained sound awareness and insisted using the implant all day. The category of auditory performance scores (CAP) showed in 84% values equal or better than CAP 4 and in 48% equal or better CAP 5 with CAP 7 being the best value.

**Conclusion:**

ABI is a safe and successful surgical procedure for restoration of hearing and speech in prelingual deaf children. Precious time of plasticity of the auditory pathways should be used as early and intensively as possible. Therefore in doubtful CI candidates ABI should be the primary indication.

## DGKCH: Hepatobiliary Surgery

### Malignant pediatric liver tumors – surgical treatment and outcome

(Abstract ID: 92)

M. Schulze^1^, S. Kathemann^1^, U. Dirksen^1^, E. Lainka^1^, A. Paula^1^

^1^*Universitätsklinikum Essen*

**Background:**

Liver tumors in children are a rare condition. Mainly they can be classified as hepatoblastomas, hepatocellular carcinomas or very rare enteties as for example mesenchymal hamartomas wich are considered semimalignant as they can proceed to sarcoma of the liver. All of these have an indication for resection or if not resectable (except for hamartomas) livertransplantation. We thoroughly studied the possibility for resection in all cases before proceeding to liver transplantation.

**Materials and methods:**

At our institution we analyzed surgical porcedures of children with liver tumors. Between January 2012 an Junly 2018. There were 17 cases of malignant pediatric liver tumors undergoing surgery. Patients age was between 0 and 14 years. 11 patients suffered from hepatoblastoma, 3 from hepatocellular carcinoma and 3 from mesenchymal hamartomas.Liver transplantation was perfomed in 4 cases, 2 of them hepatoblastomas, 2 hepatocellular carcinomas. Liver transplantation was living donor transplantation for 3 out of 4 patients.

**Results:**

The children with hepatoblastomas were younger mean age 2.2 years (8m, 3f) mean age for hepatocellular carcinoma was 6.6.years (2m 1f) and 4.6 for mesenchymal hamrtomas (2m 1f). Mesenchymal hamaroma cases were totally resected as anatomical liver resection. One HCC patient was resected and the other two underwent liver transplantation. 9 of 11 hepatoblastoma cases were resected primarily or after downstaging according to SIOPEL protocol and two underwent liver transplantation. Overall survival was 100%, progression free survival up to this point equally at 100% and morbidity was low including minor woundinfections (n=2), ascites (n=3) and pleural effusion (n=1). Mean intraoperative blood transfusion needed for anatomical resection was 0.5 untis, for transplantation 1.2 untis.

**Conclusion:**

Surgical resection or liver transplanation in our intitution is safe. In times of organ shortage, especially for pediatric recipients, we managed to avoid liver transplantation in 13 of 17 cases. If liver transplantation was necessary we perfromed living donor liver transplantation in all except of one case.

### Treatment and outcome of the patients with rhabdomyosarcoma of the biliary tree. Experience of the Cooperative Weichteilsarkom Studiengruppe (CWS)

(Abstract ID: 101)

C. Urla^1^, S. Warmann^1^, M. Sparber-Sauer^2^, A. Schuck^3^, I. Leuschner^4^, T. Klingebiel^5^, G. Blumenstock^1^, E. Koscielniak^2^, J. Fuchs^1^

^1^*Universitätsklinikum Tübingen*

^2^*Klinikum Stuttgart*

^3^*Klinikum Ingolstadt*

^4^*Universitätsklinikum Schleswig-Holstein, Kiel*

^5^*Universitätsklinikum Frankfurt am Main*

**Background:**

Biliary rhabdomyosarcoma (RMS) is the most common biliary tumor in children. The management these patients pose unique challenges due to the rarity of this tumor entity and its critical location at porta hepatis, which makes a radical resection very difficult to achieve.

**Materials and methods:**

A retrospective chart analysis of patients suffering from biliary RMS who were registered into three different CWS trials (CWS-96, CWS-2002P, and SoTiSaR registry) was carried out.

**Results:**

17 patients (12 female, 5 male) with a median age of 4.3 years fulfilled the inclusion criteria for this analysis. The median follow-up was 42.23 months (10.7-202.5). The 5-year overall (OS) and event free survival (EFS) rates were 58% (45-71) and 47% (34-50). Patients >10 years of age and those with alveolar histology had the worst prognosis (OS 0%). The patients with botryoid histology had an excellent survival (OS 100%) compared to those with non-botryoid histology (OS 38%, 22-54, p=0.047). Tumor size had no influence on survival (p=0.351). There was no statistical significant difference in survival rates of patients treated with complete primary or secondary resection compared to those who underwent incomplete primary or secondary resection and were irradiated (p=0.67).

**Conclusion:**

Biliary RMS is a rare tumor entity. Positive predictive factors for survival are age <= 10 years and botryoid histology. Tumor size had no influence on survival.

### Diaphragmatic hernia following pediatric liver transplantation: Case series of four cases with a focus on recurrence

(Abstract ID: 129)

L. S. Waldron^1^, M. F. Berger^2^, O. J. Muensterer^1^, M. Cortes Cerisuelo^3^, E. Lurz^2^, M. Guba^2^, D. J. Lo^4^, J. F. Magliocca^4^

^1^*Universitätsmedizin der Johannes Gutenberg-Universität, Mainz*

^2^*Uniklinik München*

^3^*Kings College London, London*

^4^*Emory University School of Medicine Atlanta*

**Background:**

Diaphragmatic hernia (DH) is a rare but potentially life-threatening complication of liver transplantation (LT) in children. Although literature is scarce, it appears that early detection and prompt surgical management is the cornerstone of successful outcome. Here, we present four cases, two of which showed recurrence after what appeared to be proper surgical correction. Additionally, we review the current literature focusing on recurrence of this rare complication.

**Materials and methods:**

This case series represent a patient cohort derived from a joint effort between three major transplant programs. Ten previously published patients with DH from these centres were excluded.

**Results:**

We identified four children who suffered from DH following liver transplant. All were either reduced grafts or SPLIT grafts. All were left lateral segments. All hernias occurred on the right. In all cases, small bowel was herniated into the right chest. Two children presented with nausea and vomiting, the other two were asymptomatic. There was variation of surgical technique upon repair, hinting a possible risk factor for recurrence. Two hernias recurred within six months of original correction and could be corrected successfully with additional surgery. While one child presented with nausea and vomiting upon recurrence, the other was asymptomatic. All children were well with working grafts one year following DH repair. There were no other associated complications.

The pathophysiology of a diaphragmatic hernia remains unclear with a reported incidence rate between 2.0 and 2.8%. However, the literature suggests the incidence is underreported. Potential causal risk factors include the pressure differential between the abdominal and thoracic cavities, delayed healing caused by the diaphragmatic movement, the thin musculature of the diaphragm, direct surgical trauma through the use of diathermy during the hepatectomy, and a hypothetical physiologic site of minor resistance (locus minoris resistentiae) which is clinically irrelevant as long as the native liver is in situ. Implied quantitative risk factors include young age, use of left lateral segments, high graft-to-recipient body weight ratio (GBWR)- grafts, primary closure of the abdomen, small recipients, previous surgery, injury to the diaphragm and the type of immunosuppression. These risk factors may provide the criterion for a guideline for early detection.

**Conclusion:**

We report an additional four cases of DH after pediatric liver transplantation, two of which recurred within six months. Among other factors, surgical technique may play a role as a risk factor for recurrence. Furthermore, we encourage surgeons to report cases more thoroughly in hope to raise awareness of the potential risk to patients. If tissue appears thin during the primary operation, prophylactic plicating reinforcement of the right diaphragmatic dome may be advisable.

### Decision Making and Treatment Approach in Children with Congenital Portosystemic Shunts

(Abstract ID: 206)

B. F. B. Mayer^1^, L. Sieverding^1^, E. Sturm^1^, S. Warmann^1^, J. Fuchs^1^

^1^*Universitätsklinikum Tübingen*

**Background:**

Congenital portosystemic shunts (CPSS) are short cuts between the portal venous and the systemic circulation. They are rare anomalies that result from developmental abnormalities of the vitelline veins and the inferior vena cava (IVC). CPSS contain a risk for encephalopathy and liver dysfunction when a large ratio of blood is diverted to the systemic circulation. Main goal of treatment is to avoid portal hypertension and liver transplantation. The aim of our study was to evaluate children with CPSS and analyse the decision making process for the therapeutic approach as well as treatment course and outcome.

**Materials and methods:**

We retrospectively evaluated 6 children with CPSS who were treated at our institution between 2004 and 2018. Four of them had an Abernethy type CPSS, 1 had an intrahepatic shunt connecting the right portal to the right hepatic vein and 1 child presented with a patent ductus venosus connecting the portal vein to the IVC. All patients received multiple slice CT or MRI scans with contrast medium as well as angiography for diagnostic work up. The decision making process was based on an interdisciplinary conference for every patient.

**Results:**

The mean age of patients (3 girls and 3 boys) at primary investigation was 34 months (range 3.5 - 104.5 months). Two patients (1 with an Abernethy type CPSS and 1 with an intrahepatic shunt) received interventional occlusion using an Amplatzer vascular plug. In these 2 patients good liver function and sufficient portal venous flow was observed during follow-up. One patient with a patent ductus venosus underwent ductus banding. In this child sufficient portal venous flow and good liver function were observed at discharge. Follow-up care was provided at a foreign hospital. One patient with an Abernethy type CPSS received surgical shunt ligation. The abdomen was temporarily closed andthe patient monitored at the paediatric intensive care unit. The abdomen was closed permanently 48 hours later after lactate levels had decreased and a constantly sufficient portal venous flow was observed. In this patient good liver function and sufficient portal venous flow was observed during follow-up visits. One patient with an Abernethy type CPSS did not receive interventional occlusion, since the shunt was too short. The patient is currently scheduled for surgical banding. One patient did not receive interventional or surgical treatment because of a hypoplastic portal system. This patient is currently scheduled for liver transplantation. All 4 patients who underwent surgical or interventional treatment received anticoagulatory treatment throughout the follow-up period. Mean follow-up was 27 months (range 6 - 57 months).

**Conclusion:**

Interventional or surgical occlusion, ligation or banding are possible treatment approaches for CPSS in children and infants. Preoperative evaluation of portal venous flow and determination of the type of CPSS is crucial to determine if there is portal patency. In case of portal patency, interventional or surgical treatment should be considered. If there is no portal patency, conservative management is initiated in order to delay liver transplantation as far as possible.

## DGKCH: Research in pediatric surgery

### NETs markers are predictive for pediatric appendicitis: A pilot study in mice and humans

(Abstract ID: 87)

M. Klinke^1^, S. Klohs^1^, N. Mokhaberi^1^, A.-L. Schacker^1^, M. Trochimiuk^1^, L. Pagarol Raluy^1^, K. Reinshagen^1^, M. Boettcher^1^

^1^*Universitätsklinikum Hamburg-Eppendorf, Hamburg*

**Background:**

Appendicitis is one of the most frequent emergencies in pediatric surgery. In children diagnosis can be complex and relies inter alia on laboratory values like CRP and leucocytes. These biomarkers are unspecific and have low predictive values. Neutrophiles and NETosis (a key effector function of neutrophils) are an essential component of the immune defense against bacterial infections. The aim of this pilot study was to establish a murine model of appendicitis and to evaluate NETs markers to diagnose appendicitis in mice and humans.

**Materials and methods:**

For the study 6 weeks old C57BL/6 mice were used and advanced appendicits was induced using a modified CLP (caecal ligation puncture, figure 1) procedure. In control animals only a laparotomy was performed. In all animals blood work, CRP, cell-free DNA (cfDNA), neutrophilen elastase (NE), myeloperoxidase (MPO) and citrullinated Histion H3 (H3cit) was assessed very 24 hours. Additionally, in 5 children with histology confirmed appendicitis and in 5 matched chontrols without appendicitis the same markers were evaluated.

**Results:**

In total 20 mice (12 appendicitis- and 8 control group) were utilized. All animals survived the procedure until day 5. All mice developed an advanced form appendicitis with focal peritonitis. In mice and humans NETs markers correlated significantly with appendicitis.

**Conclusion:**

The modified CLP procedure is an excellent model for advanced appendicitis. NETs markers appear to be an excellent biomarker of appendicitis and should be validated in large prospective clinical study.

**Picture:**
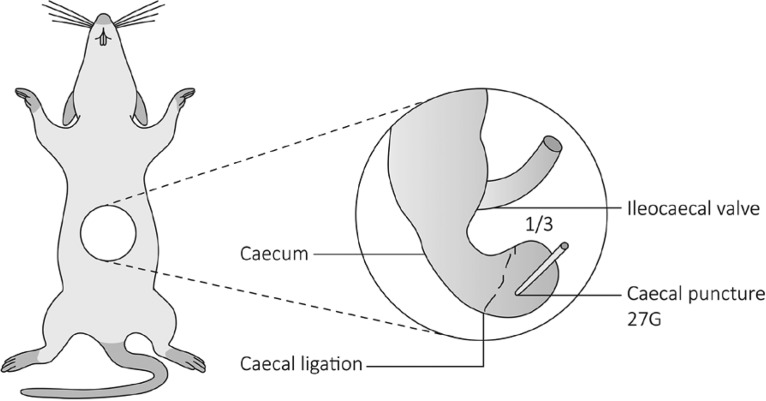
Abbildung 1: Schema der modifizierten CLP-Prozedur zur Induktion einer fortgeschrittenen Appendizitis.

### The markers of neutrophil extracellular traps (NETs) are predictive for appendicitis in children: A prospective, multicenter cohort study

(Abstract ID: 111)

S. Klohs^1^, N. Mokhaberi^2^, M. Trochimiuk^1^, A. Schacker^2^, M. Klinke^1^, L. Pagerols-Raluy^1^, K. Reinshagen^1^, M. Boettcher^1^

^1^*Universitätsklinikum Hamburg-Eppendorf, Hamburg*

^2^*Asklepios Klinik Altona, Hamburg*

**Background:**

The appendicitis is one oft the most frequent emergencies in pediatric surgery. In children the diagnosis can be complicated. The usual biomarkers (C-reactive protein, leucocyte count) are unspecific with low positive predictive values. In a pilot study we identified markers of neutrophile activation and neutrophile extracellular traps (NETs) as predictors of appendicitis. The aim of the current study has been an evaluation of these markers in comparison to the markers used to date, in a large collective of children with suspected appendicitis.

**Materials and methods:**

The prospective, multicenter cohort study has been approved by the ethical committee of the Hamburg Medical Association (PV5459). In all children with suspected appendicitis we determined leucocytes, CRP, extracellular DNA (cfDNA), neutrophile elastase (NE), myeloperoxidase (MPO) and citrulinized histone H3 (H3cit) in blood samples taken at the time of first clinical presentation. Children with immunosuppression e.g. after transplant surgery or with autoimmune disease were excluded from the study.

**Results:**

We included a total of 97 children in the study. The study reveals a significant correlation between NET markers and appendicitis and its severity respectively

**Conclusion:**

The markers of neutrophil activation and release (NETosis) are excellent biomarkers for appendicitis in children. The markers can be determined easily and cost-effectively and should be validated in other prospective multicenter studies.

### Challenges and obstacles in paediatric surgical research in Germany and potential ways for future improvement

(Abstract ID: 248)

C. Oetzmann von Sochaczewski^1^, O. J. Muensterer^1^

^1^*Universitätsmedizin der Johannes Gutenberg-Universität, Mainz*

**Background:**

Contemporary paediatric surgical research in Germany is experiencing a crisis: While research is conducted at a few notable productive centres, German paediatric surgery units have overall become less effective in terms of internationally recognized academic output, compared to other countries. We aimed to quantify the deficit and identify contributing factors.

**Materials and methods:**

The number of publications in the "Journal of Pediatric Surgery" in the last three years were compared between Germany, the Netherlands, and Israel and correlated with the number of paediatric surgical units and practicing paediatric surgeons in the respective countries. We chose the Netherlands and Israel as comparators because they both have a much smaller paediatric surgical community, but have been among the top ten contributing countries in the "Journal of Pediatric Surgery" in the last three years. To account for a possible regional mismatch due to possible different journal preferences between the different countries, we also analysed the 25th to 27th volume of the "European Journal of Pediatric Surgery". We conducted interviews among our colleagues to identify challenges and obstacles that may hamper academic productivity.

**Results:**

In Germany, 769 paediatric surgeons work at 86 departments. Cumulatively, they published an average of less than four papers per year in the "Journal of Pediatric Surgery" over the last three years (0.005 manuscripts/(year*surgeon)). In the Netherlands, there are 24 designated paediatric surgeons working in seven departments. These together published an average of eight papers per year (0.334 manuscripts/(year*surgeon), 8th rank of contributing countries)). Israel has an active paediatric surgery workforce of 60 paediatric surgeons distributed among 18 departments. They together achieve to publish four papers per (0.067 manuscripts/(year*surgeon), 10th rank of contributing countries). Within the last three years, the annual numbers of manuscripts were more wavering between the years. Nevertheless, German paediatric surgeons had a score between 0.009 and 0.016 manuscripts/(year*surgeon) and were thus way behind their comparators, which scored 0.017 and 0.033 manuscripts/(year*surgeon) in Israel and 0.083 0.33 manuscripts/(surgeon*year) in the Netherlands. Possible obstacles for academic productivity identified during our interviews were insufficient non-performance-based funding, regulatory requirements, complex animal welfare laws and necessity to take part in multiple (FELASA-B/C) courses, and increasing methodologic demands by reviewers, especially in statistical evaluation and the complexity of techniques. While advanced statistical methods are often required, statistical counselling is a rare resource at many institutions.

**Conclusion:**

German paediatric surgery does not reach its full potential when it comes to scientific productivity. Institutions should provide more incentives for academic output. Departments should consider providing protected research time to their staff and trainees and implement a standardised curriculum to provide necessary knowledge.

### Association of a microsatellite locus in the IL11 promoter with Hirschsprung´s disease

(Abstract ID: 310)

M. Haase^1^, A. Schulze^1^, I. Kemnitz^1^, G. Fitze^1^

^1^*Universitätsklinikum Dresden*

**Background:**

The most frequent known genetic alterations in Hirschsprung´s disease (HSCR) are in genes of the Ret signaling pathway or in genes that are important for neural crest differentiation. In a recent publication of an Asian population, SNPs (single nucleotide polymorphisms) in the IL11 gene were associated with HSCR. We wanted to find out, whether this also holds true for a German population.

**Materials and methods:**

Genomic DNA was extracted from 103 HSCR patients and 128 healthy control persons. The DNA sequence was determined by Sanger Sequencing. Data were analyzed using Cochrane Armitage´s trend test, chi-square test and Fisher´s exact tests.

**Results:**

In our population, there was no correlation with IL11 SNPs and the frequency of HSCR. However, we found a microsatellite locus in the IL11 promoter/enhancer region with variable repeat numbers of GT or CT dinucleotides. Shorter GT repeats were more frequent in the control population. The occurrence of more than 7 GT repeats were significantly more frequent in the HSCR population. In addition, more than 7 GT repeats were more frequently observed in Long-segment HSCR.

**Conclusion:**

To our knowledge, this is the first description of an association of a promoter microsatellite locus with HSCR. While the pathomechanism linking HSCR with IL11 is not resolved, one might speculate that IL11 protein expression may have an influence on an inflammatory phenotype of HSCR. Further studies are necessary to determine functional consequences of the GT-repeat length on the expression of the IL11 protein.

### Short and long term outcome for patients with surgical removal of sacrococcygeal teratoma in newborns and infancy

(Abstract ID: 349)

M. Ai^1^, V. Schellerer^1^, M. Besendörfer^1^

^1^*Universitätsklinikum Erlangen*

**Background:**

Sacrococcygeal teratomas (SCT) are a relatively uncommon tumor affecting neonates, infants and children with an incidence of 1:27000. This study evaluates the short and long term outcomes of newborn and infant cases with sacrococcygeal teratoma removal. We examined the physical and neurological impairment affecting these patients, as well as risk factors for adverse outcome.

**Materials and methods:**

A total of 19 patients underwent surgical procedure between July 2001 and September 2018 in our pediatric surgery department. We gathered information from the patient medical records, postoperative medical interviews, and performed physical examinations. We used this information to evaluate the status and the extent of the impairment of the patients. The patients received follow up treatment in according to the MAKEI recommendations for duration of 10 years.

**Results:**

19 patients had surgical procedure through our department due to sacrococcygeal teratoma (male: n = 5, female n = 14). SCT are more common in females with a 3 to 4:1 ratio, in our research we had a 3:1 ratio. Pre-operative assessment included prenatal ultrasound (n = 19), prenatal MRI (n = 5), neonatal MRI (n = 8). Prenatally 11 patients were diagnosed with SCT, while 6 patients were diagnosed postnatally. In 2 cases the time of diagnosis is unclear. Most of our patients presented with both internal and external pelvic location of the tumor. The distribution of our Altman classification was: 15% Altman I (n=3), 42% Altman II (n=8), 10% Altman III (n=2), 10% Altman IV (n=2), 15% Altman unknown (n=3). The surgery was performed at the age of diagnosis which was on average the 2nd day of life (range: 0 to 13 day of life, in 2 patients 5 to 6 months of age). The weight median was by 2800g with a range of 2570 g. Out of the 19 patients, 11 tumors were completely removed with R0 surgical resection (n=11), 3 tumors with R1 (n=3) and 5 tumors with RX (n=5) surgical resections. Histological findings included predominantly chondroid (n = 12) and neuroectodermal (n = 10) parts. The maturity level was evaluated: 33% GC 0 (n = 6), 11% GC 1 (n = 2), 16% GC 2 (n = 3), 5% GC 3 (n = 1), 33% GC unknown (n = 6). Post-operative complications included surgical site infections (n = 5), bowel perforation (n = 1), and post- operative mortality (n = 2, due to underlying diseases). 11 of the patients have long term follow ups through our hospital; all 11 patients are satisfied with the outcome and the scars. Post-operative long- term complications include: sphincter dysfunction (n = 2), neurogenic bladder dysfunction (n = 1), neurogenic bowel dysfunction (n = 1), functional leg length difference (n = 1), recurrent urinary tract infections (n = 1). There was one case of tumor recurrence with a second surgical removal. The oncological follow ups continued for 10 years after surgery, some are ongoing. One patient was diagnosed with embryonic carcinoma, received chemo therapy and has since passed away.

**Conclusion:**

Sacrococcygeal teratoma is a heterogeneous entity of the germline tumors and the prognosis is significantly better for patients who had R0 surgical resection and low GC scores. The patients and the parents were satisfied with the results of the surgical removal of the tumor. Follow ups are necessary to determine future reoccurrence of the tumor.

### Increasing incidences of cholecystolithiasis in pediatric surgery: A single center review

(Abstract ID: 438)

S. Diez^1^, V. Schellerer^1^, A. Hoerning^1^, M. Besendoerfer^1^

^1^*Universitätsklinikum Erlangen*

**Background:**

Compared to adult patients, the incidence of cholecystolithiasis is rare in pediatric patients within Europe. However, with increasing incidence of obesity in children, more and more children and adolescents present with gallstones at our institution. Our objective was to review the risk factors, surgical timing, complications and outcomes of cholecystolithiasis and cholecystectomy at our institution.

**Materials and methods:**

Patient data were obtained by reviewing the records of all patients with conducted cholecystectomy between January 2010 and September 2018. Clinical and surgical data were assessed, focusing on concomitant morbidities, timing of surgical planning and intra- and postoperative conditions. 23 children and adolescents were included in this retrospective study.

**Results:**

We conducted cholecystectomy due to cholecystolithiasis in 23 patients during 2010 and 2018. Among all included 15 female (65%) and 8 male patients (35 %), the median age was 14 years (range 8-17 years). At diagnosis, 48% of the children were obese with a median BMI of 23.3 kg/m2 (range 12.9-36.6 kg/m2). Concomitant diseases were seen in 48% (n=11) with a high incidence of spherocytosis (22%, n=5). One patient presented with symptoms under chemotherapy of Burkitt lymphoma, one patient presented with unilateral infantile cerebral palsy and in one case, continuous parenteral nutrition was necessary due to short bowel syndrome after gastroschisis and intrauterine volvulus. Additionally, permanent medication due to cardiac defects, bronchial asthma and hypothyreosis could be observed. All patients suffered from classical symptoms, biliary pancreatitis was initial symptom in 5 patients (22%). In 9 cases, conservative treatment to avoid surgical approach was primary conducted over a period of 309 days (range 181-636 days) with clinical observation and intermittent symptomatic therapy. Therapy could not achieve sustained effect, so that indication for surgery was given. Specialized treatment, such as resolution of gallstones by ursodeoxycholic acid, was applied in 4 patients (17%, this alternative treatment was not applied before 2014). In 4 cases of biliary pancreatitis, ERCP was conducted for papillotomy and stone extraction prior to surgery, but could not replace surgical treatment as multiple gallstones were confirmed. Laparoscopic surgery was preferred as surgical approach (91%, n=21). In one case, surgery had to be converted to open cholecystectomy due to previous complex abdominal surgery and consecutive adhesions. There were no postoperative complications to observe. Median time to discharge was 4 days (range 3-11 days). Median time to follow up was 31 months.

**Conclusion:**

Incidence of cholecystolithiasis and consecutive cholecystectomy in children and adolescents is on the rise, as obesity in children and adolescents is increasing. Alternative conservative treatment should represent an option to reduce symptoms. Due to tendency of chronical inflammatory processes and high percentage of concomitant diseases in children and adolescents, time for optimal surgical intervention is difficult to detect in children. Laparoscopic excision remains treatment of choice in symptomatic cholecystolithiasis.

### A highly dysregulated circular RNA profile in lungs from patients with congenital diaphragmatic hernia could serve as a potential prenatal biomarker

(Abstract ID: 507)

R. Wagner^1^, A. Jha^2^, S. Kahnamoui^2^, L. Ayoub^2^, D. Patel^2^, T. H. Mahood^2^, C. D. Pascoe^2^, J. H. Gosemann^1^, M. Lacher^1^, R. Keijzer^2^

^1^*Universitätsklinikum Leipzig*

^2^*University of Manitoba, Winnipeg*

**Background:**

Approximately, 1 in 3000 babies is born with Congenital Diaphragmatic Hernia (CDH), a disease characterized by a hole in the diaphragm and underdeveloped lungs. Morbidity and mortality of CDH are determined by the degree of lung hypoplasia and pulmonary hypertension. Epigenetic factors are involved in the pathogenesis. Circular RNAs are powerful epigenetic regulators of gene expression, especially in embryonic development. However, their involvement in abnormal lung development is still unknown. We hypothesized that circular RNA profiles of human CDH lungs are dysregulated and aimed to determine their potential as biomarkers for CDH in the future.

**Materials and methods:**

Lung tissues for CDH (n=6) and healthy controls (n=6) were obtained for mid-pregnancy cases and end-pregnancy cases from deceased subjects. After total RNA isolation circular RNA expression was profiled via circular RNA microarray (Arraystar Inc., Rockville, MD, USA). In depth statistical data analysis was performed with R Studio. Pathway analysis was performed using KEGG and Ingenuity Pathway Analysis (Qiagen).

**Results:**

CDH lungsshowed a highly altered circular RNA profile compared to lungs from healthy controls for both, mid - and end - pregnancy cases. Partial least squares discriminant analysis segregated clearly into two independent clusters. VIP-score analysis revealed the most important circular RNAs responsible for the profile alterations. In total, 16 circular RNAs were significantly altered (Fold change > 1.5; p-value < 0.05) at mid-pregnancy and 35 circular RNAs at end-pregnancy.

**Conclusion:**

Circular RNA profiling of human hypoplastic CDH lungs and healthy controls at two different developmental time has the potential to differentiate between CDH and controls.Further investigation of these candidate circular RNAs and correlation with the clinical course of patients with CDH might reveal potential biomarkers for the early prenatal diagnosis of CDH.

### Laparoscopic-assisted hydrostatic Reduction of Intussusception in pediatric patients with unsuccessful hydrostatic Desinvagination

(Abstract ID: 558)

A. Alsweed^1^, L. Müller^1^, J. Leonhardt^1^

^1^*Klinikum Braunschweig*

**Background:**

Intestinal intussusception is a serious abdominal emergency in infancy and Childhood, with a high mortality and morbidity rate if not diagnosed and treated early and correctly. The accepted initial Therapy now is the reduction of the Intussusception hydrostatic or pneumatic.

We present our experience in the pediatric surgery in Braunschweig bei using Laparoscopic-assisted hydrostatic Reduction in Patients who previously having unsuccesful hydrostatic reduction in Department of pediatrics.

**Materials and methods:**

From February 2018 to September 2018, we operated 7 patients ( 3 girls, 4 boys, aged between 18 Months and 8 years ) with ileocoloic invaginations, in whom prior hydrostatic desinvagination had failed. We want to present the operation technique used and report our experience.

**Results:**

In 5 cases desinvagination was achieved using laparoscopic procedures and Ringer lactat enema. In 2 Patients, conversion to a non-laparoscopic technique was required during the procedure. In those two cases pathological lead points (Burkitt lymphoma, hetrotopic Pancreas) were found afterwards.

**Conclusion:**

If the conservative approach failes, laparoscopic desinvagination is good, safe und with minimal manipulation of the bowels. It helps to desinvaginate those patients.

### Bladder augmentation with Lyoplant^®^ – First experimental results in rats

(Abstract ID: 637)

T. Meyer^1^

^1^*Universitätsklinikum Würzburg*

**Background:**

Congenital defects of the urinary bladder (micro- or contracted bladder, bladder exstrophy) propose a challenging problem for pediatric surgeons and urologic surgeons. Even at full exhausting all conservative treatment options, in a large number of cases there is an irreversible renal dysfunction and even lead to chronic renal failure and the need for kidney transplantation. To protect kidney function, a bladder augmentation with bowel is necessary. There are many problems and complications on this current standard method with the augmentation of intestinal as an unphysiological material, such as recurrent urinary tract infections or bladder stone formation. For this reason, a number of substitutes for the implementation of a bladder augmentation has been proposed, but without success.

**Materials and methods:**

We implantated a biocompatible collagen mesh (Lyoplant®) in a bladder defect rat model (n = 16). After 6 weeks, the abdomen was reopened the initial implant and the bladder were resected for histological and immunhistochemical examination.

**Results:**

There were no technical difficulties in implantaning all the different materials. There were no wound healing, wound infections and no herniation. The implants presented macroscopically cell infiltration and neovascularization. Adhesions were found in the animals rarely. The light microscopic analysis showed cell infiltration.

**Conclusion:**

In summary, Lyoplant® is very suitable for experimental bladder augmentation in rats.

## DGKCH: Pediatric Oncology: Metastasis Surgery

### Interdisciplinary approach reveals cerebral sarcoma abdominally metastasised via VP-shunt

(Abstract ID: 318)

E. Ammer^1^, C. Bock^1^, I. Kühnle^1^, I. Metz^1^, F. Kahl^1^

^1^*Universitätsmedizin Göttingen*

**Background:**

Abdominal metastases of cerebral tumors via VP shunts are rare but have been described several times in the past. In the presented case of a 4-year-old girl the VP shunt had been installed to reduce intracerebral pressure (ICP) caused by bifrontal hygromas. This treatment may have disguised symptoms of increasing ICP caused by an aggressively growing cerebral sarcoma. It only became clinically apparent after the VP shunt had occluded. To further evaluate the shunt dysfunction a sonography of the abdomen was induced. It revealed that by this time the tumor had already metastasised into the abdominal cavity. The subsequent laparoscopy, laparotomy and trepanation to gain tissue verified the suspicion of a cerebral sarcoma which had metastasized via the VP shunt.

A VP-shunt facilitates distant seedling form the cancer origin. Close surveillance and interdisciplinary interaction are compulsory preconditions for tumor diagnosis and treatment.

## DGKCH: Pediatric traumatology: Fractures of lower extremity - Femur

### Spica cast immobilization in the management of pediatric unstable diaphyseal femur fractures

(Abstract ID: 132)

A. Herzog^1^, T. Wirth^1^, F. Fernandez^1^

^1^*Klinikum Stuttgart*

**Background:**

With an estimated prevalence of under 1%, unstable diaphyseal femur fractures represent one of the less common skeletal traumatic injuries in childhood. However, the preference regarding the optimal management strategy in infants and toddlers, particularly the application of spica cast immobilization, has always been involved in heated debates in the field of pediatric orthopedic trauma.

While there are currently no official recommendations on this issue, the purpose of this retrospective review is to evaluate the clinical outcomes of unstable diaphyseal femur fractures that were primarily treated through spica cast immobilization.

**Materials and methods:**

We have reviewed 62 cases involving traumatic, non-pathological, unstable diaphyseal femur fractures in children aged under 4 years old at the time of the injury that were treated in our department in a period of 10 years between 2008 and 2017. All cases were primarily treated through spica cast immobilization, and depending on the initial alignment, either with or without an adjuvant reduction.

The demographics of the study cohort spread between 2 days and 3.9 years of age (1.9 ± 0.8). All children that included in this study suffered from an unstable diaphyseal femur fracture, where 54 cases involved a spiral fracture while the other 8 cases involved a transverse fracture. In 17 cases the initial alignments were non-displaced and the initially displaced alignments in the other 45 cases varied from 10 degrees valgus to 44 degrees varus (10 ± 9.9).

**Results:**

Within the subcohort that involved cases with initially displaced alignments, an adjuvant reduction was performed prior to the application of spica cast when the initial alignment was either more than 5 degrees valgus or more than 10 degrees varus. All spica casts were applied under general anesthesia within 24 hours after admission. The average hospitalization lasted between 1 and 3 days (1.5 ± 0.6). The duration of spica cast immobilization lasted between 2.1 and 4.9 weeks (3.3 ± 0.6). No severe complications occurred during the immobilization period, especially no skin breakdown, nerve or vascular damages were observed upon cast removal. Nonunion was observed in none of the cases. One case of secondary displacement occurred upon consolidation and this case was further treated through flexible intramedullary nailing. The alignments upon consolidation ranged between 8 degrees valgus and 28 degrees varus (7.8 ± 7.2). 26 cases with malunion in terms of over-limit malalignments were scheduled with a follow-up examination between 28.4 and 171 weeks (64.5 ± 46.3) after consolidation. At the time of the follow-up examination, over-limit malalignments up to 14 degrees varus (7 ± 5) could still be observed in 5 cases, so that a further follow-up examination was scheduled between 1 and 6.2 years (3.5 ± 2.4) after consolidation, where a long-leg standing radiograph was performed, which showed a leg length discrepancy between 8 and 15 mm (10.6 ± 2.6) with the initially injured leg being at disadvantage.

**Conclusion:**

In conclusion, spica cast immobilization has been proven to be a valid treatment option for traumatic, non-pathological, unstable diaphyseal femur fractures in infants and toddlers.

Temporary persistence of over-limit valgus or varus malalignments upon consolidation usually diminish spontaneously within a reasonable time frame with midterm leg length discrepancy generally under the tolerable upper limit.

Comparably shorter hospital stay and better subjective comfort are further advantages of spica cast immobilization.

### Under-4-year old patients treated with overhead extension for isolated femoral shaft fracture – lessons learned concerning parents‘ satisfaction

(Abstract ID: 390)

V. Schütz^1^, F. Wagner^1^, J. Hubertus^1^

^1^*Uniklinik München*

**Background:**

Conservative options for the treatment of femoral shaft fractures remain the standard for patients under the age of 4 years. This includes primary spica cast treatment (SCT) and/or overhead extension therapy (OHE). In order to improve the satisfaction of the parents, we have designed a study investigating possible influencing factors on parents’ contentment.

**Materials and methods:**

Forty-two children under the age of 4 years treated with initial OHE for isolated femoral shaft fracture were analyzed. Our standard treatment protocol implies change to SCT after 10 days and subsequent discharge from hospital care. X-rays were analyzed for angular deviation, leg length shortening and dislocatio ad latum and for correction of the dislocation during the course of treatment. A pseudonymonized questionnaire was sent to the parents in order to evaluate their experiences during the hospital stay and follow-up.

**Results:**

On the day of accident the nature of the fracture was explained "understandable and in detail" to 73.3% of the parents. X-rays were demonstrated in 86.7% cases. 23.3% stated that "many questions were not answered". About half of the parents were informed about the different therapeutic options. About two thirds of parents stated a sufficient to very good pain management at the day of admission. 70% of parents noticed behavioral abnormalities during treatment. Additionally, 63.3% reported treatment-specific problems. This included decubitus and the necessity to change the extension bandages in the majority of cases.

When asked for the reasons of non-satisfaction, most reasons related to communicational problems. 86.7% of parents were generally satisfied with the therapy outcome and most (80%) were content with the type of therapy ("very good", "good" and "satisfactory"). Nevertheless, 13.3% of the families complained about the condition of the leg after treatment, 10.0% of patients needed furthermore therapy due to decubitus or non-satisfactory healing and one patient attracted attention because of walking problems. In addition, 6.7% of families stated that the expended effort regarding extension therapy was not worth the good therapy outcome. Nevertheless, 73.3% would choose OHE again.

When performing Spearman correlation (ρ) we found a correlation between high general satisfaction and the parents, which felt well informed about the nature of the fracture. We found no correlation between high satisfaction and the information given about advantages and disadvantages of the different treatment options. More importantly, there was a strong correlation with a high general satisfaction and the fact that mentioned problems during the medical rounds were addressed appropriately (ρ=0.552; p= 0.002). Same was found for the aspect of communication with the healthcare professionals (ρ=0.528; p = 0.003) and the care provided by the nurses (ρ = 0.501; p = 0.005).

**Conclusion:**

Besides the significant correlation between high general satisfaction and sufficient information about the nature of the fracture we found personal and respectful contact to be a major influence factor for parents’ satisfaction. A good valuation of the medical rounds, the communication with healthcare professionals and the care provided by the nurses did affect the contentment of the families significantly.

## DGKCH: Urological problems with anorectal malformations in children

### Proximal urethral stenosis in patients with anorectal malformation

(Abstract ID: 634)

T. Klein^1^, L. Gindner^1^, A. Ekamp^1^, T. M. Boemers^1^

^1^*Kinderkrankenhaus Amsterdamer Straße, Kliniken der Stadt Köln gGmbH*

**Background:**

Proximal urethral stenoses in male patients with anorectal malformation are rare. They can be congenital or acquired, latter caused by a damage of the urethra during the surgical reconstruction of the anorectal malformation. Depending on the degree of the congenital urethral stenosis it is associated with bladder distention, vesicoureteral reflux, hydronephrosis, renal dysplasia, oligohydramnios, and other deformities.

**Materials and methods:**

A retrospective study of all male patients with anorectal malformation and congenital or acquired proximal urethral stenosis treated in our department between January 1st, 2005 to December 31st, 2018 was performed. The focus of this study was set on the different diagnostic and surgical approaches as well as the course of follow-up.

**Results:**

Two patients with a significant acquired proximal urethral stenosis after external surgical repair of the ARM were identified. In one case we reconstructed the urethra by an anastomosis of the urethra and the bladder neck. In the second patient the parents refused the urethral reconstruction and the child still has a cystostomy.

Furthermore, five patients with congenital proximal urethral stenosis were identified. We performed a dilatation and stenting of the urethral stenosis in one patient, a Sachse internal urethrotomy in another patient, a perineal transfer of the urethra in two cases, and in one case a surgical repair of the urethra by a perineal approach.

**Conclusion:**

Congenital as well as acquired proximal urethral stenosis require a precise diagnosis by cystoscopy and cystography. Depending on the exact location and the length of the stenosis a different procedure is necessary. Based on our case series the deciding diagnostic steps and different treatment alternatives are explained.

## DGKCH: Minimally invasive surgeryin children: My worst case

### Intrathoracic pectus bar dislocations after implementing a short-bar technique

(Abstract ID: 465)

J. Gödeke^1^, S. Rohleder^1^, O. J. Muensterer^1^

^1^*Universitätsmedizin der Johannes Gutenberg-Universität, Mainz*

**Background:**

In 2015, we adopted a short bar technique for minimal-invasive repair of pectus excavatum. Since then, we experienced three delayed intrathoracic bar dislocations. This case series describes the particular circumstances, salvage techniques, and discusses possible future prevention strategies.

**Materials and methods:**

The patients underwent uneventful MIRPE using a short bar technique (10" bar, one or two lateral stabilizers). They were completely back to normal life when they suddenly developed chest pain. In patient 1 (17y, male), the bar slid out of the stabilizer on the left side due to material break of the locking rivet 10 months postoperatively, which led to intrathoracic bar dislocation. In patient 2 (20y, male) Intrathoracic dislocation of the bar and attached stabilizer was diagnosed on the right side (figure 1) 13 months post MIRPE. Patient 3 (15y, male) had an uneventful course and good cosmetic result, with no symptoms. Left intrathoracic displacement was noted on the preparatory chest radiograph performed routinely before bar removal.

**Results:**

The first 2 patients were reoperated without further complications. In patient 1, we performed thoracoscopic bar replacement in original MIRPE technique. There was no obvious organ laceration. In patient 2, the displaced bar was removed via a thoracoscopic-assisted open approach according to patient´s choice, accepting partial correction of the deformity. Slight atelectasis of the right lower lung lobe resolved with forced inspiration. In this patient, intensive intercostal stripping was noted as a potential underlying reason for the bar dislocation. In the third patient, the bar was removed under thoracoscopic guidance without incidence. We had not seen cases of intrathoracic bar displacement in our previous cohort using long bars.

**Conclusion:**

We hypothesize that short bars predispose to intrathoracic dislocation due to diminished extrathoracic bearing surface and support, and increased more concentrated forces. This may lead to intercostal stripping and subsequent intrathoracic migration of the bar. In order to minimize the tendency to erode through the intercostal space, wider stabilizers, less bar curvature, and stronger fixation of the stabilizers to the ribs may be advisable. Also, stabilizers should be placed medially, close to the point of chest wall penetration, and bars should be long enough for the free end to cover at least two ribs.

**Picture:**
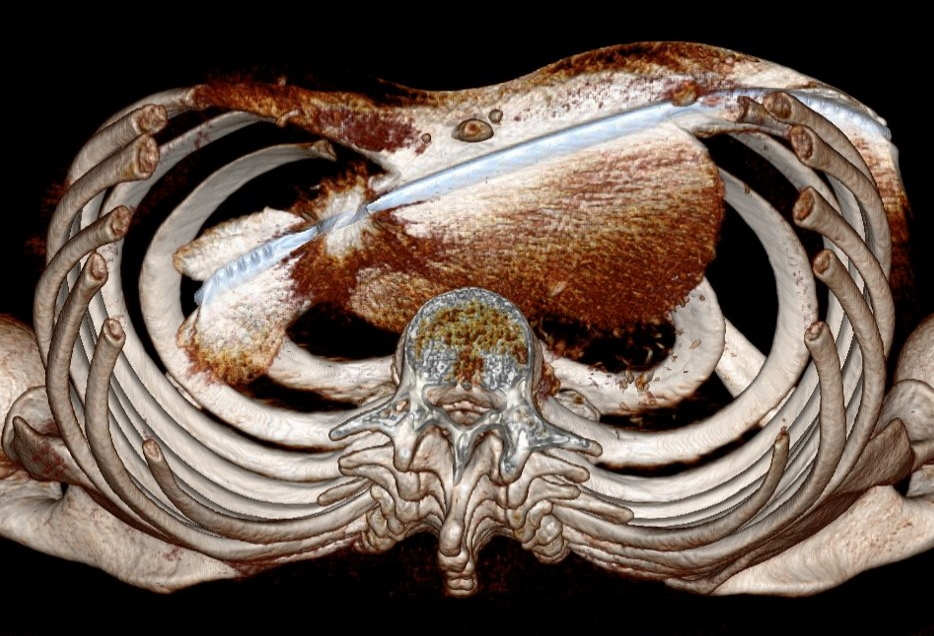
Three dimensional computed tomography reconstruction of intrathoracic pectus bar displacement on the side with the stabilizer attached, compressing the heart and lung.

### Neodymium magnets – dangerous toys everywhere

(Abstract ID: 590)

O. H. Diez^1^, B. I. Diez-Mendiondo^1^, G. Nonnenmacher^1^, N. Laizer^1^, T. S. Bott^1^, U. Mehlig^1^, S. Loff^1^

^1^*Klinikum Stuttgart*

**Background:**

Supermagnets are on the rise. Especially Neodymium magnets are very popular because they are very small and offer a heavy adhesive force. We find these magnets in toys, as Mini-Magnets, ornamentation articles, speakers, working tools and locking covers of tablets e.g..

Usually there is no problem by swallowing one magnet if it’s not too big. But children sometimes swallow more than one magnet at different times. This can lead to significant gastrointestinal problems.

There are several reports of bruise accidents and gastrointestinal perforations caused by Neodymium magnets because of the very high adhesive force they have.

We report of a 3 year old boy which had swallowed 2 supermagnets in a short time and went to the ER because of vomiting.

**Materials and methods:**

After establishing the diagnosis of 2 foreign bodies at the esophago-gastric junction by X-ray this child was treated operatively. First we tried to get out the magnets by endoscopy.

**Results:**

The two magnets were sticked together in the junction by sticking esophagus and cardia together. Because of the very high adhesive force of the supermagnets it was impossible to get the magnets apart. We had used the whole repertoire of endoscopic tools, but this case led into an upper median laparotomy. Even by open surgery it was very difficult to divide those Neodymium magnets. The course after operation was uneventful. The outcome was good.

**Conclusion:**

Neodymium magnets in the hands of children are dangerous. Especially when they swallow more and at different times these supermagnets. Toys should be free of these supermagnets.

## DGKCH: Perinatal surgery

### Complications of fetal intervention for sacrococcygeal teratoma

(Abstract ID: 608)

F. Obermayr^1^, G. Seitz^1^, R. M. Vahdad^1^, T. Klein^2^, T. Boemers^2^

^1^*Universitätsklinikum Marburg, Marburg*

^2^*Kinderkrankenhaus Amsterdamer Straße, Kliniken der Stadt Köln gGmbH*

**Background:**

Sacrococcygeal teratoma (SCT) represents one of the most commonly prenatal diagnosed tumor entities. Although long-term outcome of postnatal tumor resection is described to be excellent, highly vascularized teratomas can lead to high-output cardiac failure and fetal hydrops and ultimately to fetal or perinatal death. In order to improve the prognosis of these patients, fetal laser or radiofrequence ablation (RF) of tumor vessels has been proposed in order to improve survival of selected patients. While improvement of survival could be demonstrated in small case series, data about complications of prenatal intervention are limited. We describe postnatal outcome and complications of four patients who underwent prenatal laser or RF ablation of tumor vessels in SCT.

**Materials and methods:**

Demographics and clinical outcome data of patients suffering of prenatally treated SCT were analyzed retrospectively, with respect to complications of the fetal procedure, necessary postoperative surgery and functional outcome of postoperative reconstructive procedures. Data of two german pediatric surgical centers were collected.

**Results:**

Between 06/2014 an 08/2018 four patients, who underwent fetal laser or RF-ablation of tumor supplying vessels in patients suffering from SCT were admitted to two pediatric surgical centers, either for tumor resection (n=3) or for secondary reconstructive purposes (n=1). Data about prenatal cardiac failure were not available. In one patient prenatal iatrogenic tumor rupture was observed. In all patients the rectum or anus-sphincter complex was injured by fetal intervention. So far, reconstruction of the anorectum could be performed in only one patient, resulting in end-colostomy in 3 patients, and transverse loop-colostomy in another. Addition complications of fetal intervention included functional paraplegia (L4/L5) (n=1), complete unilateral destruction oft he femur head, the acetabulum and gluteal muscles (n=1) and large area skin necrosis (n=1).

**Conclusion:**

Although fetal intervention in patients with SCT might improve fetal and postnatal survival in patients suffering from high-output cardiac failure, the overall benefit of the procedure should be critically discussed, taking potential severe anatomic and functional impairment of the patients into account. In addition, insufficient data exist about potential tumor spillage, particularly in patients suffering from immature tumors.

## DGKCH: Varia

### Scrotal Incision by Acute Scrotum in Newborns

(Abstract ID: 19)

M. Sanal^1^, P. Hechenleitner^1^, O. Renz^1^, B. Häussler^1^

^1^*Medical University Innsbruck VTT Clinic, Innsbruck*

**Background:**

The aim of this report was to outpoint the possible difficulties of the scrotal approach in newborn period who underwent surgical exploration due to acute scrotum.

**Materials and methods:**

The charts of patients were reviewed retrospectively. 10 newborns underwent surgery because of acute scrotum. 7 of them was operated via inguinal incision without any additional technical problem. By 3 patients was used scrotal approach.

**Results:**

In two patients, which underwent scrotal incision occured unexpected difficulties. The first one showed small intestine protruding during the operation through the scrotal incision.The second newborn was operated 1 week after scrotal orchidopexy due to incarcerated inguinal hernia.

**Conclusion:**

The acute scrotum is defined as an emergency situation where the patient presents with acute pain, swelling and reddening of one, or rarely both hemiscrotums. This condition is much more turbulent in newborns. Urgent exploration via scrotal incision is recommended for acute scrotum. But in newborns it may be uncertainty of diagnostic workup. That is the why we prefer inguinal approcah in newborn period in case of acute scrotum.

### A pyriform sinus fistula in disguise of several acute lateral neck infections in an 8 year old girl

(Abstract ID: 584)

O. H. Diez^1^, R. Staubach^1^, S. Steiner^1^, S. Loff^1^

^1^*Klinikum Stuttgart*

**Background:**

We report a case of a left-sided pyriform sinus fistula presenting as an abscess-forming lateral neck fistula.

**Materials and methods:**

The girl presented at the age of 5 a clinical history of dysphagia and swelling of the left-sided neck. Ultrasound and MRI showed a conglomerate of lymph nodes suspicious of abscess. The abscess was split in 07/2014.

7 months later the girl had the first recurrent abscess. It was treated only with antibiotics by the ENT specialist.

The next relapse occurred in 06/2015. It started with an acute deep neck infection at the same place on the left-sided neck. The abscess was split again. Now it was on suspicion of a lateral neck fistula.

Over two years there had been no more problems. 10/2017 the girl experienced the third recurrent neck abscess on the left side. And again the abscess was split. Never there was seen a fistula. Longterm antibiotic treatment resulted in an uninflamed situation and we operated the now 8 year old girl 12/2017 on suspicion of a lateral neck fistula.

**Results:**

During operation we found a 4 cm, thin fistula running from the skin deep to the sternocleidomastoid muscle lateral to the trachea straight into the esophagus at its junction with the sinus piriformis. The fistula was completely excised, the esophagus was oversewn. Histology proved epithelial lining of the fistula.

The postoperative course was uneventful.

**Conclusion:**

Openings and recurrent abscesses at the medial border of the sternocleidomastoid muscle, otherwise typical for a lateral neck fistula, can be part of a pyriform sinus fistula originating from 3rd or 4th branchial cleft remnant which have a different course in the tissue. The surgeon must be aware of this rare possibility.

**Picture:**
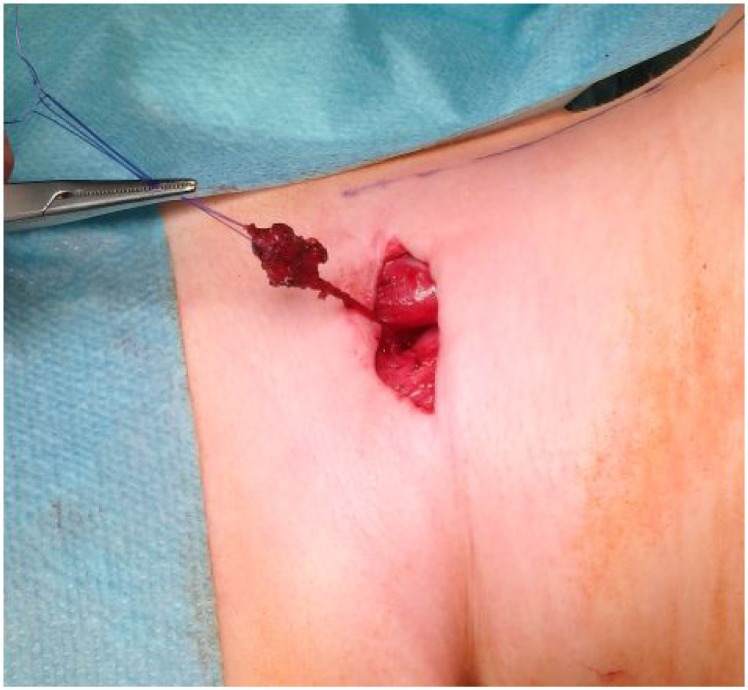
pyriform sinus

### Sonographic differentiation of complicated from uncomplicated pediatric appendicitis: Correlations between ultrasound findings and histological results

(Abstract ID: 587)

J. Reismann^1^, T. Rawolle^1^, K. Rothe^1^, M. Reismann^1^

^1^*Charité - Universitätsmedizin Berlin CVK*

**Background:**

To investigate the correlation between sonographic findings and histological results of pediatric appendicitis. In regard of creating a decision-making tool to initiate an antibiotic-only treatment for patients with uncomplicated appendicitis.

**Materials and methods:**

This is a retrospective study of 1440 pediatric patients who underwent an appendectomy in our institution between 2006 and 2016. Medical charts were reviewed for histological results and ultrasound findings. 419 patients were excluded due to missing data, secondary or elective appendectomy, oxyuriasis and sonographic examinations not performed by pediatric radiologists. The histological finding of a phlegmon was categorized as uncomplicated appendicitis, perforations and gangrenes as complicated appendicitis. Univariate and multivariate logistic regression analysis were used to investigate the association between sonographic and histological findings. Significant differences were defined by a corrected error probability of P <= 0.05.

**Results:**

Out of 568 (55.6%) male and 453 (44.4%) female patients with a mean age of 10.67 years, uncomplicated appendicitis was histologically diagnosed in 445 (43.6%) cases and complicated appendicitis in 349 (34.2%) children. 223 (21.8%) patients had negative histological results. The following ultrasound findings were significantly associated with complicated appendicitis in multivariate regression: an increased appendiceal wall diameter (OR = 1.27; P < 0.001), periappendiceal fat inflammation (OR= 1.50; P = 0.018), appendicolith (OR = 1.66; P = 0.022) and the expression of a suspected perforation (OR = 5.72; P < 0.001) of the pediatric radiologist. Periappendiceal fat inflammation showed the highest sensitivity (57.9%) and the presence of an appendicolith had the highest specificity (90.2%).

**Conclusion:**

Ultrasonography can be helpful to predict histological results regarding the patient’s treatment. Due to low sensitivity values, prospective studies with proficiently planned sonographic protocols are needed to improve the performance of ultrasound as a predictive tool.

### Pitfall of rituximab-induced intestinal perforation after heart transplantation (HTX) in a 14-year-old girl

(Abstract ID: 591)

O. Renz^1^

^1^*Tirol Kliniken GmbH - Universitätskliniken Innsbruck*

**Background:**

Post-transplant lymphoproliferative disorder (PTLD) is the name given to a B-cell proliferation due to therapeutic immunosuppression after organ transplantation. Treatment options include reduced immunosuppression, chemotherapy, rituximab, surgery and radiation, or a combination of these approaches.

**Materials and methods:**

A 14-year-old girl received a heart transplant due to dilated cardiomyopathy in June 2017. Her immunosuppressive medication consisted of glucocorticoid.

Twelve months after transplant, the girl developed PDLD on the throat and small intestine. A chemotherapy of rituximab was started.

We diagnosed intestinal perforation and performed an emergency operation. During the laparotomy, four perforations were identified at 20 cm oral from the terminal ileum. Little faeces were present, and the adjacent intestine seemed to be a suitable condition for primary anastomosis. Therefore, the patient underwent ileal resection with primary anastomosis and a high output stomata.

**Results:**

In the post-operative course, the patient was managed jointly by a surgeon and paediatric oncologist A. Her condition was managed by reducing immunosuppression and modifying the chemotherapy administered.

Reduction of immunosuppression and administration of endoxan was started. The patient experienced complete remission four months after the first surgery.

**Conclusion:**

PTLD is a potentially fatal complication after heart transplantation and encompasses a broad spectrum of tumours, ranging from benign polyclonal lymphoid proliferation to high-grade malignant lymphoma. In this case, rituximab chemotherapy led to a potentially fatal situation, which could only be prevented by surgery and revision of the therapy.

### Entrappment of median and ulnar nerve after open diaphyseal forearm fracture in a 7 year old boy

(Abstract ID: 621)

C. Matissek^1^, F.-M. Häcker^1^, A. Mack^1^, T. F. Krebs^1^

^1^*Ostschweizer Kinderspital, St. Gallen*

**Background:**

Diaphyseal forearm fractures are common in the pediatric population and account for 5-6% of all pediatric fractures. Nerve injury is rare, but is more often seen in open fractures. Isolated ulnar or median nerve injuries due to fracture entrappment are documented in literature but to our knowledge there is no description of a both nerves fracture entrappment.

**Materials and methods:**

we present a case of a 7-year old boy who suffered a 1° open midshaft forearm fracture after a fall from 2 meters.

**Results:**

Operative treatment included dissection of the wound at the volar and ulnar aspect of the forearm followed by closed reduction with intramedullary nailing of Radius and Ulna. Within the next three weeks he developped progressive motor and sensory deficit of the ulnar and median nerve. Sonography showed the median nerve entrapped within the fracture. Operative exploration was performed and confirmed the median nerve beeing entrapped within the radial fracture. The ulnar nerve was entrapped in callus and scar tissue around the ulnar fracture. Complete neurolysis and decompression of both nerves was performed.

**Conclusion:**

We present the rare combination of ulnar and median nerve entrappment in forearm fracture and discuss this case regarding the recent literature.

### Thyroid and parathyroid surgery in childhood – a review of a single center experience

(Abstract ID: 663)

J. Syed^1^, V. Schellerer^1^, P. Klein^1^, M. Besendörfer^1^

^1^*Universitätsklinikum Erlangen*

**Background:**

Thyroid and parathyroid surgery in childhood are rare and therefore need a specialized, coordinated, interdisciplinary management. Considering specific surgical risks, anatomical particularities and the proportion of cancer in thyroid nodules, an advanced experience level of the surgeon is strictly required.

**Materials and methods:**

We conducted a retrospective analysis of data of all children who underwent thyroid or parathyroid surgery in our department between 2000 and 2018. 49 patients between 4 and 17 years were included, 88% female (n=43) and 12% male (n=6), due to recurrence in two female patients 51 operations were performed.

**Results:**

The analyzed data show a mean age of 13 years at the day of operation and a gender distribution in favor of the female sex with 88% (n=43). Persistence of Graves' disease under conservative treatment was the most frequent cause for thyroid surgery in 35% of all cases (n=18). In 18% there was euthyroid goitre with or without nodules (n=9). 16% of the patients were suffering of a thyroid adenoma (n=8). Thyroid carcinoma (CA) was diagnosed in 12% (n=6), hereof 83% papillary CA (n=5) and 17% medullary CA (n=1). In 8% Hashimoto´s thyroiditis was the cause for thyroid surgery (n=4). A spindle cell tumor in a patient with infantile myofibromatosis was diagnosed in 2% (n=1). In a total of 10% diseases of the parathyroid glands required surgery (n=5), hereof 60% parathyroid adenoma (n=3) and 40% parathyroid hyperplasia (n=2). Two patients underwent a second operation due to recurrence - in one patient a left sided struma nodosa occurred 19 months after hemithyroidectomy of the right lobe, so left sided hemithyroidectomy was required, and in one patient three hyperplastic parathyroid glands needed to be resected four months after resection of the right lower parathyroid gland. Operative techniques contained local resections of thyroid nodules in 8% (n=4), hemithyroidectomies were performed in 20% (n=10), hereof 70% total hemithyroidectomies (n=7) and 30% near total hemithyroidectomies (n=3), of which one was performed in combination with a local resection of a nodule of the other side, thyroidectomies were performed in 63% (n=32), hereof 37,5% total thyroidectomies (n=12) and 62,5% subtotal or near total thyroidectomies (n=20), single resections of one parathyroid gland were performed in 8% (n=4) and resections of three parathyroid glands in 2% (n=1). Postoperatively persistent hypoparathyroidism occurred in 4% (n=2), both patients were suffering of infiltrative CA´s. All other cases showing hypocalcaemia only needed a temporary medication, the symptoms faded completely and laboratory findings showed standard values. A surgical side infection occurred in 2% (n=1) and a single operative wound management was performed on the 24th postoperative day. In 2% (n=1) there was a cervical hematoma without clinically relevant impairment, which faded under conservative treatment. A left sided recurrent nerve paralysis without impairment occurred in 2% (n=1) after operation of an infiltrative papillary CA.

**Conclusion:**

In addition to the rarity of thyroid and parathyroid diseases in childhood there are associated particularities related to children, which need to be considered, if surgery is required. Due to that, thyroid and parathyroid surgery in childhood should be reserved for centers with appropriate expertise and availability of all the involved specialists to guarantee a standardized, perioperative procedure and reduce complication rates significantly.

### Management and outcomes of congenital anomalies in low-, middle-, and high-income countries: Protocol for a multi-centre, international, prospective cohort study

(Abstract ID: 932)

J. Lindert^1^, K. Tafazzoli^1^, N. Ade-Ajayi^2^, A. O. Ademuyiwa^3^, E. Ameh^4^, J. Davis^2^, K. Lakhoo^5^, D. Poenaru^6^, N. Sevdalis^2^, A. Leather^2^

^1^*Uniklinik Lübeck*

^2^*King's College London*

^3^*University of Lagos and Lagos University Teaching Hospital, Lagos*

^4^*National Hospital, Abuja*

^5^*University of Oxford*

^6^*McGill University, Montreal*

**Background:**

Congenital anomalies have risen to become the 5th leading cause of death in children under 5-years of age globally, yet limited literature exists from low- and middle-income countries where most of these deaths occur.

This collaboration aims to undertake a multi-centre prospective cohort study of congenital anomalies across the globe to compare outcomes between low-, middle- and high-income countries (LM&HICs).

**Materials and methods:**

The Global PaedSurg Research Collaboration will be established consisting of children's surgical care providers from around the world to participate in the study; collaborators will be co-authors of resulting presentations and publication(s). Data will be collected on patients presenting primarily with seven congenital anomalies (oesophageal atresia, congenital diaphragmatic hernia, intestinal atresia, gastroschisis, exomphalos/ omphalocele, anorectal malformation and Hirschsprung's disease) for a minimum of 30 consecutive days between Oct 2018 - April 2019. Data will be collected on patient demographics, clinical status, interventions and outcome. Data will be captured using the online data collection tool REDCap.

The primary outcome will be all-cause in-hospital mortality and the secondary outcomes will be occurrence of post-operative complications. Chi-squared analysis will be used to compare mortality between LM&HICs and multivariate logistic regression analysis to identify factors affecting outcomes. P<0.05 will be deemed significant. Ethical approval will be sought from all participating centres. Funding has been granted by the Wellcome Trust.

**Results:**

The study aims to be the first large-scale, geographically comprehensive, multi-centre prospective cohort study of a selection of common congenital anomalies across the globe to define current management and outcomes, aid advocacy and global health prioritisation, and inform future interventional studies aimed at improving outcomes.

